# Outcomes of diffuse large B-cell lymphoma in elderly patients—real-world experience from a middle-income country setting

**DOI:** 10.3332/ecancer.2021.1242

**Published:** 2021-06-03

**Authors:** Chandrayee Sarker, Vivek S Radhakrishnan, Payal Mandal, Jeevan Kumar, Saurabh Bhave, Rimpa Achari, Debdeep Dey, Indu Arun, Zameer Latif, Neeraj Arora, Deepak Mishra, Mammen Chandy, Reena Nair

**Affiliations:** 1 Department of Clinical Hematology, Tata Medical Center, 14 Main Arterial Road (EW), Newtown, Kolkata 700160, India; 2 Department of Radiation Oncology, Tata Medical Center, 14 Main Arterial Road (EW), Newtown, Kolkata 700160, India; 3 Department of Pathology, Tata Medical Center, 14 Main Arterial Road (EW), Newtown, Kolkata 700160, India; 4 Department of Hemato Pathology, Tata Medical Center, 14 Main Arterial Road (EW), Newtown, Kolkata 700160, India; a https://orcid.org/0000-0001-9484-5669

**Keywords:** diffuse large B-cell lymphoma, elderly, anthracyclines, rituximab, dose intensity

## Abstract

**Background:**

Diffuse large B-cell lymphoma (DLBCL) is the commonest subtype of lymphoma in the elderly and poses unique challenges in this group of patients. There is a need for more information on real-world outcomes across economic disparities.

**Methods:**

Electronic Medical Record of 3,087 lymphomas (>18 years) were evaluated retrospectively, of which 842 (27%) patients were ≥65 years. Two hundred and twelve patients who were ≥65 years received first line treatment for DLBCL between May 2011 and Dec 2016. Demography, clinical features, associated co-morbidities, first line treatment outcomes and hospital costs were analysed. Patients were followed up till March 2020.

**Results:**

The median age at presentation was 71 years. Gender ratio was 2.5:1. 38% patients presented with early-stage disease, 37% with low and low-intermediate International prognostic index, 49% with nodal disease. One or more co-morbidities were present in 58%. The commonest extra nodal site was gastro-intestinal (29%). Two-thirds of the patients presented with non-Germinal centre B subtype. The overall response (OR) to treatment was 72.5%. Patients who received anthracycline-based therapy (*n* = 124) and rituximab-based therapy (*n* = 159) had a median progression free survival (PFS), not reached and 47.0 months, respectively, versus 10 months and 7.9 months, respectively, for patients receiving non-anthracycline and non-rituximab therapies. At a median follow-up of 24 months, the 5-year overall survival and PFS are 44% and 41%, respectively, for the entire cohort.

**Conclusions:**

DLBCL is a curable lymphoma in elderly patients with standard anthracycline and rituximab-based therapies. Improvement in outcomes largely depends on social and financial support to complete the scheduled treatments.

## Introduction

Diffuse large B-cell lymphoma (DLBCL) is the most frequent subtype of lymphoma in the elderly [1]. Numerous studies have described lower response, shorter disease-free survival and overall survival (OS) rates [2–5] in older patients. ‘Elderly’ is defined by the WHO as anyone above the age of 65 years [6]. The average life-expectancy in India is 66.6 years for men and 69.4 years for women [7]. Management of these patients remains a challenge due to the following reasons: presence of significant co-morbidities and frailty, delayed access to care, significant socioeconomic dependence, under-representation in prospective clinical trials, scanty literature and lack of an evidence-based approach, aggressive disease biology, higher toxicity, compromised dosing and early curtailment of treatment [5, 8–10].

When paired for histological and clinical characteristics of lymphoma, the survival is shorter in the elderly patients compared to younger [11, 12]. The difference remains significant after adjustment for non-lymphoma related deaths. Aggressive disease biology and a higher incidence of the non-Germinal centre B (non-GCB) phenotype are attributed to the poor outcomes [13, 14]. Elderly patients are at an increased risk of toxicity of chemotherapy. Very significant changes in weight, nutritional status and easy fatigability, accompanied by poorly controlled comorbidities have been observed [5, 9]. The shorter survival could also relate to a trend in the prescription of weaker treatments assumed to be better tolerated [2]. Most clinical trials have focused on patients between 60 and 80 years with DLBCL [15, 16]. An assessment of co-morbidities and a comprehensive geriatric assessment (CGA) are valuable tools in assisting physicians [17]. However, these scales are difficult to implement in a busy clinical practice.

Current therapeutic options for elderly DLBCL have largely been extrapolated from treatment results in younger patients, predominantly due to an under-representation of the elderly in large clinical trials. Most clinicians in a busy practice setting with concerns of comorbidities, frailty and disproportionate toxicity in the older patients tend to reduce dose and use less intensive or abbreviated regimens. These practices potentially impact outcomes in the elderly. CHOP-R (cyclophosphamide, adriamycin, vincristine and prednisolone along with Rituximab) remains the most widely used and standard-of-care regimen. In patients with cardiac insufficiency and other associated co-morbidities, alternative regimens include CEOP-R (cyclophosphamide, etoposide, vincristine and prednisolone along with Rituximab) or BR (Bendamustine and Rituximab), but no definitive ‘best regimen’ has been defined.

We conducted a retrospective audit of DLBCLs ≥ 65 years, treated at our institute. This helped us look at the real-world outcomes in a middle-income country setting, identify the challenges in practice and look at potential solutions for the future.

## Methodology

Between May 2011 and December 2016, 3,087 patients (>18 years) with a diagnosis of lymphoma were registered at our centre, 1,617 (52%) received frontline therapy for their disease, 101 (3%) were treated for relapsed or refractory disease, 299 (10%) were treated elsewhere and followed up at our centre and 1,068 (34%) registered for a second opinion. Patients with less than four visits in the outpatient clinic with no definite treatment prescribed at our centre, were considered as second opinion seekers and were not analysed in this audit. Eight hundred and forty-two patients (27%) were ≥65 years. Two hundred and twelve treatment naive DLBCL patients were considered evaluable for first line treatment. The audited data has been approved for presentation by the institutional review board (Hospital Ethics committee). A waiver for consent was granted.

### Disease assessment

Details collected at diagnosis included clinical presentation, medical history, co-morbidities, laboratory tests, treatment and related toxicity. This information was obtained from the hospital electronic medical records (EMRs). In patients reporting post-biopsy, the histopathological diagnosis was reviewed at the centre (subject to slide and block availability) prior to start of therapy. DLBCLs were classified into germinal centre B cell (GCB) or non-GCB based on an immune histochemistry approach using the Han’s algorithm [18]. Tests for Proliferative index Ki-67, bcl-2 and immunohistochemistry (IHC)/fluorescent in situ hybridization (FISH) C-myc were undertaken to differentiate DLBCL from Burkitts’ and Burkitt-like lymphoma.

Clinical variables recorded from the EMR included age, gender, Eastern co-operative oncology group performance status (PS), fever (>38.6°C), weight loss (>10% of body weight in 6 months) and Ann Arbor stage. Pre-existing co-morbidities were also recorded. Echocardiography was done at baseline for all patients, and at interim/end of treatment in most patients. As part of staging evaluation, positron emission tomography-computerized tomography (PET-CT) or CT imaging of the thorax and abdomen along with bone marrow biopsy was done. Cerebrospinal fluid (CSF) cytology at diagnosis was done for patients with high risk of central nervous system (CNS) involvement. Laboratory test results recorded were absolute blood counts, creatinine, albumin and lactate dehydrogenase (LDH). Hypoalbuminaemia was defined as an albumin level <3.5 gm/dL. The cut off for haemoglobin was 10 gm/dL. The International prognostic index (IPI) on the basis of the following criteria, i.e., PS (0–1 or >1), Ann Arbor stage (localised versus extensive), extranodal site (0, 1 versus 2) and LDH (normal versus upper normal value) was calculated for each patient [11]. Response assessments were done mid cycle and at the end of treatment. The end of treatment response is reported in the audit. Follow-up information of each patient was obtained from the EMR records, or by contacting the patient or family. Reasons for death have been classified into three groups as follows: progressive disease (PD), treatment toxicity and other causes.

### Treatment, response and safety

In elderly DLBCL patients, the choice of therapy was dependent on the patient’s general condition, co-morbidities, financial constraints and social support. Treatment decisions were physician determined as in a ‘real-world’ situation and Rituximab was added to the regimen depending on financial feasibility of the patient. Pre-phase chemotherapy with steroids alone or with cyclophosphamide and vincristine (CVP) was given to patients with PS > 2 at presentation, high LDH and advanced stage disease. The Group 1 received low dose oral chemotherapy (steroids, cyclophosphamide and etoposide), along with supportive care in the frail elderly patients. Group 2 received non-anthracycline combination chemotherapy (CVP/CEOP+/−R, B-R) when physicians considered cardiotoxicity as a limiting factor for patients with adverse reports on echocardiography evaluation and/or prior significant cardiac history. Patients with CNS lymphoma received methotrexate and cytarabine based therapy with or without whole brain radiotherapy. Group 3 received multiagent regimens with anthracycline in standard doses (CHOP+/−R), as infusional therapy [etoposide, prednisolone, vincristine, cyclophofamide, adriamycin-Rituximab (EPOCH-R)] or as miniCHOP-R. Early-stage patients received four cycles of chemotherapy followed by radiotherapy, if they achieved an early response. Advanced stage disease was treated with six cycles of therapy followed by radiotherapy to the site of initial bulky tumour or for partial response (PR). Inclusion of rituximab in the chemotherapy regimen increased over time with improvements in patient access programmes, and availability of multiple affordable generic brands.

Dose intensity (DI) was calculated for patients who received anthracycline-based or etoposide-based (in the CHOP like regimen, instead of anthracycline) therapy. The DI was calculated as mg/m^2^/week of adriamycin (or etoposide) and cyclophosphamide. Impact on progression free survival (PFS) was calculated for DI ≥ 80%, and <80% planned dose. The efficacy of treatment was assessed according to the National Cancer Institute-sponsored International Working Group criteria established in 1999 [12]. Treatment-related toxic effects leading to hospitalisation and reported in the EMR were analysed. Patients in remission were censored at last follow-up. Patients with no follow-up information (physical/telephonic) after progression were considered deceased.

### Statistical analysis

OS was calculated as the time from registration to the last follow up or death. The predictive covariates of survival analysed for internal validation of the cohort were gender, stage, IPI and previous co-morbidities. PFS data was computed by the Kaplan–Meier analysis method and survival curves were compared using the log-rank test.

### Hospital costs

The hospital costs were calculated under the headers of investigations, procedures, imaging, pharmacy, blood bank services and infection management. These included costs during hospitalisation and outpatient treatment and follow-up till the date of audit analysis, in March 2020. The pharmacy costs include costs of all chemotherapy, immunotherapy, supportive treatments, antimicrobial therapy for infections and costs of medications for management of other intercurrent illness. The blood bank expenses included costs of any component therapy administered during the treatment and follow up. This cost-analysis was restricted to Group 3, where the treatment intention was essentially curative. The median costs were calculated separately for private and the subsidised category of patients, and therapeutic subgroups of CHOP+/−R, miniCHOP+/−R and EPOCH-R.

## Results

The clinical characteristics of elderly DLBCL patients are summarised in Table 1. The median age was 71 years for the cohort. The gender ratio was 2.5:1. The median duration of symptoms was 3 months (range: 15 days to 18 months) with 38% patients presenting with early stage (1 and 2), 37% with low IPI and 49% with nodal disease. The common extra nodal sites were gastro-intestinal (29%), head & neck (16.5%), genitourinary (15.5%), lung (12.5%), bone (9.5%), CNS (7%) and others (para-vertebral, breast, soft tissue, etc.). DLBCL subtypes on morphology included *de novo* DLBCL in 178, high grade DLBCL in 23, transformed follicular or low-grade lymphoma in 6, Grey zone in 3 and one each of cyclin D1 positive, plasmablastic rich and T-cell rich B-cell lymphoma. The cell of origin (COO) on block review (by Hans-algorithm) was available for 144 patients: 47 were GCB (32%) and 97 non-GCB (67%).

Staging evaluation by PET-CT was available in 57 patients (27%), and CT thorax and abdomen in the remaining. Information from bone marrow biopsy was available in 129 (60%) and CSF cytology in 17 (8%). A repeat PET scan for mid treatment response evaluation was done in 60 patients (28%) and an end of treatment PET was done in 89 (41%). The remaining patients had evaluation done with CT scans.

Data from age, stage, extranodal site and IPI were well validated for OS, as shown in Table 2. While age, stage and IPI had an impact, extra-nodal presentation did not impact the OS. GCB subtype had a median survival of 29 months and non-GCB subtype 14 months.

### Treatment efficacy

Pre-phase chemotherapy was administered in 114 patients (53%) to stabilise the patient(s) prior to the planned chemotherapy protocol. Eight patients did not receive further therapy. Patients received a median of six cycles (range: 0–6) of chemotherapy. The distribution of treatments according to the predefined subgroups is summarised in Table 3. Only 50% of the patients received Rituximab in the initial period (2011–2013) of this study. This increased to 90% of the patients in the years following 2013. Complete response (CR) was observed in 115 patients (54%), PR in 31 (15%) and stable disease in 8 (4%), after first line chemotherapy. The OR rates for treatment Groups 1, 2 and 3 were 7%, 72% and 81%, respectively. The CR rates were 3.5%, 47% and 68%, respectively. Thirty-one patients (14.6%) had PD on first line treatment. Progression on treatment was seen in 37%, 18% and 8% in groups 1, 2 and 3, respectively. Twenty-seven (14%) patients could not be evaluated for response. This was due to early treatment-related mortality in seven patients (Infections in five patients, haemorrhage and tumour lysis in one each); severe morbidity leading to early cessation of treatment in five patients; treatment discontinuation due to financial constraints in seven patients; undetermined cause for early discontinuation in four and continuation of treatment elsewhere in four patients.

**Figure 1.**
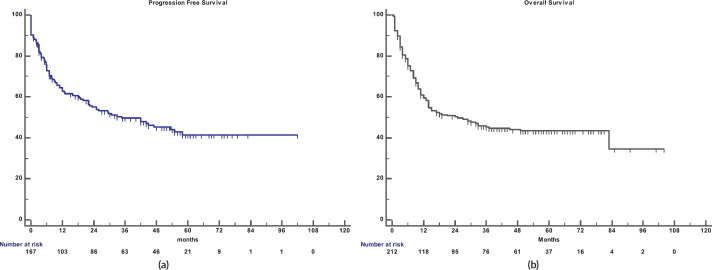
(a): PFS for Elderly DLBCL patients. (b): OS for the elderly DLBCL patients.

**Figure 2.**
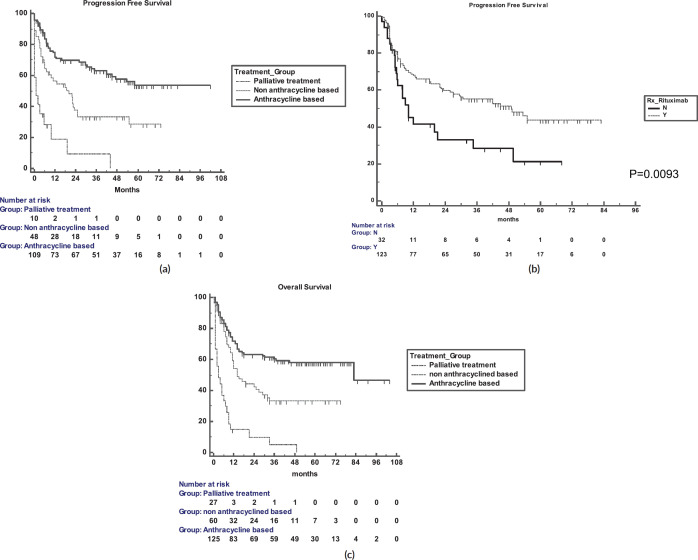
(a): PFS for the three treatment groups. (b): PFS in the Rituximab and no-Rituximab groups. (c): OS for the three treatment groups.

**Figure 3.**
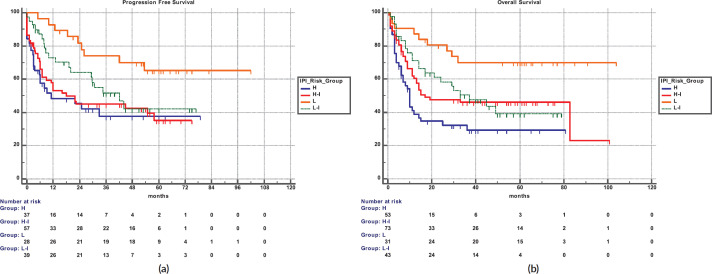
(a): IPI and PFS. (b): IPI and OS.

The 5-year PFS for the entire cohort is 41% (Figure 1a), while the 5-year OS is 44% (Figure 1b). The median PFS for treatment groups 1, 2 and 3 are 1 month (95% CI: 0.0–44.0), 20 months (95% CI: 6.1–25.0) and not reached, respectively (*p* < 0.0001) as shown in Figure 2a. The median PFS for patients receiving anthracycline was not reached as compared to 10 months (CI: 4.9–20.0) in patients who did not receive anthracycline based therapy (*p* < 0.0001). The median PFS for patients receiving Rituximab was 47 months (95% CI: 25.0–58.0) and 7.9 months (95% CI: 5.0–20.0) for those who did not receive Rituximab (*p* < 0.0001), as shown in Figure 2b. The median PFS was not reached in 49 patients who received a DI ≥ 80%, and 42 months in 85 patients who received DI of <80% (95% CI: 16.0–58.0), respectively (*p* = 0.1388). The median OS for the treatment groups 1, 2 and 3 was 3 months (95% CI: 1.0–7.0), 14 months (95% CI: 11.0–29.0) and 83 months (95% CI: 37.0–83.0), respectively (*p* < 0.0001), as shown in Figure 2c. IPI was prognostic for survival outcomes, both OS and PFS, in an expected and significant pattern, as shown in Figures 3a and b.

### Co-morbidities

Data available and recorded in the EMR showed one or more co-morbidities in 125 patients (58%). In the cohort of patients where co-morbidity records were available, hypertension was the commonest co-morbidity in 89 (71%), followed by diabetes in 60 (48%), hypothyroidism in 16 (12%), chronic obstructive lung disease in 13 (10%) and ischaemic heart disease in 33 (26%). Blood borne virus screening reports were available in 159 patients. Hepatitis B surface Antigen (HBsAg) reactive status was observed in eight patients, HCV in five and a confirmed HIV in one. The co-morbidity status of the patient did not have an impact on the outcomes, as shown in Table 2.

### Complications

There was paucity of information regarding grade 1–2 toxicity in the EMR. In this background, only the serious adverse events (requiring hospitalisation) and changes in medication due to complications were analysed. Eighty-six patients (40%) were hospitalised for grade 3 or 4 toxicity. Nine (12%) patients were hospitalised more than once. The median days of hospitalisation were 6 (range: 1–40). Febrile neutropenia was the commonest cause of hospitalisation (*n* = 29) followed by respiratory tract infection in 12, gastrointestinal infection in 4, urinary tract infection in 4 and Cytomegalovirus, Herpes zoster, Hepatitis and acute cholecystitis in 1 patient each. Vincristine induced subacute intestinal obstruction resulted in hospitalisation in eight patients, hyponatraemia in three and tumour lysis in one. Haemorrhage occurred in four patients (one at the tracheostomy site and three gastric haemorrhage) and were managed conservatively. Two patients required surgical intervention for intestinal obstruction and perforation. Four patients developed ischaemic heart disease, with congestive cardiac failure in two and acute myocardial infarct in two. Peripheral neuropathy grade 3, requiring drug withhold and/or replacement of vincristine to vinblastine was recorded in four patients.

### Hospital costs

In the group 3 patient cohort, the median expenditure on investigations, procedures and imaging services combined was Rs 109,500 ($1500). In the same group, the median expenditure on pharmacy costs was Rs 181,500 ($2500), while Blood bank and infection management costs accounted for Rs 20,000 ($ 250), from diagnosis to the last follow up. In patients who received standard therapy with CHOP, the cost of investigations inclusive of procedures and imaging was Rs 80,000 ($1000), pharmacy costs Rs 138,900 ($2000) and supportive care costs on account of blood products and infection management were Rs 15,000 ($200). Among patients receiving miniCHOP and EPOCH, the respective median costs of investigation (procedure and imaging included) were Rs 158,875 ($2200) and Rs 385,810 ($5000), Pharmacy costs Rs 322,500 ($4300) and Rs 422,500 ($5600), blood products and infection management costs were Rs 20,000 ($250) and Rs 66,500 ($850).

## Discussion

In the treatment of DLBCL, CR rates reported with CHOP chemotherapy in the older patients have been 50% in those aged 65–75 years, and 40% in those over 75 years of age. Median remission duration was 16 months and cure rates were in the range of 25%–30%. The outcomes of patients receiving standard dose anthracycline-based chemotherapy were similar to younger patients but associated with higher toxicities and mortality from intercurrent illnesses [19–22]. The feasibility of delivering full dose CHOP therapy with myeloid growth factor support has been demonstrated earlier in older patients [23].

In the last two decades, R-CHOP administered every 3 weeks has been established as the standard-of-care for older fit patients, accompanied by appropriate supportive care [15, 24]. In the GELA study, treatment naïve DLBCL patients, aged 60–80 were randomised to receive eight cycles of R-CHOP or CHOP therapy. The CR rates were 76% and 63%, with a 10-year PFS of 36.5% and 20%, and 10-year OS at 43.5% and 27.6%, respectively. Deaths due to second cancers, late relapses and other causes were comparable [25]. The Ricover-60 Trial examined the role of six or eight cycles of CHOP-14 or R-CHOP-14. Outcomes were not better for eight cycles of chemotherapy [26]. Further, the LNH03-6B study compared R-CHOP-21 and R-CHOP-14 [27]. At a median follow up of 56 months, neither the 3-year PFS (60% versus 62%) nor the 3-year OS (69% versus 72%) differs in the respective treatment arms. In the very elderly, the frail or in those unfit for R-CHOP-21, alternative lower-intensity regimens, accompanied by appropriate supportive measures and frequent toxicity monitoring can be more than palliative in outcomes, and could potentially add meaningful quality/quantity of life. These approaches have been explored in phase 2 trials and include liposomal doxorubicin, Rmini-CHOP, infusional regimens, R-CVP or replacement of Adriamycin with mitoxantrone, gemcitabine or etoposide [28–30]. The Bendamustine Rituximab combination has been examined in small series and has demonstrated an OR of 69% with a favourable toxicity profile [31, 32].

In our study with 212 patients and a median age of 71 years, 58% (*n* = 125) presented with one or more comorbidities, 63% presented with stage III or IV disease, 51% demonstrated extra-nodal disease and over 60% had a prognostic score of high-intermediate or high. COO classification for subtyping DLBCL was available in 144 patients, of which 67% had a non-GCB subtype and 32% GCB. This reflects the disease profile that presents to a tertiary care centre in a middle-income Asian country. A CGA at diagnosis was not undertaken, and frailty was determined predominantly by eyeballing, PS assessment, comorbidity review and clinician group decisions. This highlights the constraints of applying comprehensive CGA scales in a busy centre. They are time consuming, require caregivers and physicians to enter information and a dedicated speciality service in most situations. A more realistic scale would be PS, age, comorbidities and an easier-to-use scoring system like the FIL Criteria [33]. A male referral bias in our dataset is consistent with experience from other Indian tertiary care hospitals [34].

Fifty-three percent (53%) patients received some form of pre-phase therapy. In practice, many centres administer an initial ‘pre-phase treatment’ with prednisolone for 7 days alone or with vincristine 1 mg which decreases the first cycle effects of deep neutrophil nadir, longer neutropenia duration, tumour lysis and therapy associated mortality with R-CHOP therapy [32]. In group 1, patients with poor performance and advanced disease were treated with oral chemotherapy or steroid monotherapy, and the response rate was 7% with no long-term survivors. Most patients in the groups 2 and 3 received curative treatment. The availability of generic rituximab [31] and its addition to an anthracycline based regimen significantly improved CR, PFS and OS. Though high response rates were observed with anthracycline based regimens, adverse events requiring hospitalisation were also high (40%). The most common causes for hospitalisation were Febrile neutropenia and infection. This was especially seen in patients treated with EPOCH regimen for high grade and advanced stage DLBCLs, and in the very elderly patients treated with miniCHOP. These patients needed more frequent interventions such as central line insertions, increased supportive care requirements during treatment and higher hospitalisation rate for adverse event management.

Patients labelled as ‘second opinion-seekers’ could potentially belong to a group which lack the ability to pursue treatment for social and economic reasons. Access to healthcare, good supportive care (including intensive care), good nutrition and affordability broadly determine outcomes in this setting [35]. In an out-of-pocket healthcare system, socio-economic dependence on other earning family members, overall fitness and mobility are significant determinants of outcomes.

This retrospective study enabled us to understand the true magnitude of the challenges we experience with the management of elderly DLBCL patients at our centre. This has enabled us to streamline the care of elderly patients by adopting the following approaches: careful co-morbidity and PS assessment, cross-consultation with speciality colleagues regarding comorbidity status prior to the start of definitive therapy, use of pre-phase therapy and use of age and fitness appropriate treatment regimens based on a multi-disciplinary team discussion for all patients. CGA is now increasingly considered an essential tool in determining treatment pathways for elderly patients [36]. The availability of an easy-to-use and quick tool, especially in centres without a dedicated geriatrician or a geriatric care service, remains a challenge. Robust single domain geriatric assessment tools and Mobile health technology may provide potential solutions [37, 38]. The role of supportive care interventions for symptom control and improvement of physical, emotional, and cognitive function among elderly cannot be under-emphasised. Continuous assessment during the course of treatment and follow up has a positive impact on overall outcomes [36].

## Conclusions

DLBCL is a curable disease in the elderly by using age and comorbidity appropriate treatments with chemo-immunotherapy. Management of the elderly patient needs close attention to medical, socio-economic, and emotional support in order to complete treatment and achieve desirable outcomes. The care of the elderly remains a challenge in developing economies.

## Funding

No relevant funding.

## Conflicts of interest/competing interests

RN has received research grants, advisory board fees as well as Speaker fee from Cipla, Fresenius Kabi, Johnson and Johnson, Mylan, Novartis and Dr Reddy’s Laboratory. VSR reports advisory fees (institutional) and non-financial institutional support from PFIZER, institutional grants and non-financial support from INTAS Pharmaceuticals, institutional grants from NATCO Pharmaceuticals, institutional grants from ROCHE, institutional grants from BMS, institutional grants and non-financial support from CIPLA Pharmaceuticals, institutional grants from EMCURE, personal fees (institutional) from ASTRA ZENECA, non-financial institutional support from Dr Reddy's Laboratories, outside the submitted work. Other authors declare no relevant conflicts of interest with respect to the submitted work.

## Availability of data and material (data transparency)

Yes, on a reasonable request to the corresponding author.

## Code availability (software application or custom code)

Not applicable.

## Authors’ contributions

RN conceived and lead the idea. RN, VSR and CS wrote the manuscript, contributed to the design and editing the manuscript. Patient data acquisition was undertaken by CS, and costing data by PM. Data analysis was undertaken by RN, and literature search by VSR and RN. DD, IA, ZL, NA and DM contributed to laboratory studies and edited the manuscript. VSR, RN, DD, IA, ZL, NA, DM, SB, JK, RA and MC reviewed the manuscript. Manuscript finalised by RN and VSR.

## Ethics approval

Institutional Review Board (Ethics Committee) waiver. Retrospective study with no patient personal identifiers.

## Consent to participate

Not applicable, waived.

## Consent for publication

Not applicable.

## Figures and Tables

**Table 1.** Clinical characteristics of the 212 DLBCL patients at presentation.

	Number # 212	Percent	Oral therapy or BSC # 27	Non-anthracycline # 61	Anthracycline based # 124
**Age** median (range)	**71 (65–95)**		75 (67–95)	72 (65–89)	70 (65–87)
<75	147	69	13	44	90
≥75	65	31	14	17	34
**Gender**: male	151	71	19	47	85
Female	61	29	8	14	39
**Stage:** 1	31	15	5	11	15
2	49	23	5	13	31
3	59	28	7	18	34
4	73	34	10	19	44
**IPI score^a^:** low	31	16	2	7	22
Low-intermediate	43	21	3	15	25
High-intermediate	73	34	8	24	41
High	53	26	5	16	32
**Extranodal**: nil	105	49	15	32	58
61	82	38	10	26	46
>1	25	12	2	3	20
**Sites:** gastrointestinal	38	29	5	4	27
Head and neck	21	16.5	1	9	11
Genitourinary	20	15.5	2	4	14
Lung	16	12.5	2	5	9
Bone	12	9.5	1	3	8
PCNSL	9	7	3	5	1
Other: soft tissue	12	9.5	1	1	10
**Haemoglobin^a^**: median (range)	11.3 (4.7–15.7)		10.6 (7.0–15.7)	11.7 (6.7–15.4)	11.1 (4.7–15.6)
<10.0 gm/dL	44	21	6	9	29
≥10.0 gm/dL	166	79	21	50	95
**Albumin^a^:** median (range)**** <3.5 gm/dL	3.8 (2.0–5.0) 70	35	3.4 (2.1–4.4) 12	3.9 (2.0–5.0) 16	3.7 (2.1–4.8) 42
**LDH^a^**: median (range)	703 (192–12,978)		885 (368–4,291)	668 (360–3,456)	647 (192–12,978)
Normal	30	15	1	7	22
>Normal	178	85	21	55	102
**COO subtype # 144** GCB	47	32	1	11	35
Non-GCB	97	67	17	29	51

a Missing data

BSC, Best supportive care; PCNSL, Primary central nervous system lymphoma; COO, Cell of origin

**Table 2.** Outcomes of the elderly DLBCL patients.

	Median	95% confidence interval	*p* value
**OS**			
**Age group**			
<75 years ≥75 years	45.0 10.0	18.000–45.000 6.000–83.000	*p* = 0.0005
**Stage**			
I II III IV	NR 27.0 15.0 17.0	— 13.000–29.000 10.000–83.000 10.000–49.000	*p* = 0.7008
**IPI**			
Low Low-intermediate High-intermediate High	NR 33.0 15.0 10.0	— 14.000–49.000 11.000–83.000 6.000–15.000	*p* = 0.0007
**Extranodal site**			
Yes No	29.0 14.0	14.000–45.000 11.000–83.000	*p* = 0.2368
**COO**			
GCB Non-GCB	29.0 14.0	14.000–29.000 10.000–33.000	*p* = 0.0910
**Co-morbidities**			
No Yes	49.0 17.0	14.000–49.000 11.000–45.000	*p* = 0.1266
**PFS**			
**IPI**			
Low Low-intermediate High-intermediate High	— 42 18 11	— 19.000–44.000 7.000–58.000 3.600–33.000	*p* = 0.0107
**Treatment groups**			
1. Oral therapy & BSC 2. Non-anthracycline 3. Anthracycline based Overall	1.0 20.0 NR 33.0	0.000–44.000 6.100–25.000 — 20.000–58.000	**** *p* < 0.0001
**Anthracycline based therapy**			
No Yes Overall	10.0 NR 34.7	4.900–20.000 — 21.900–58.000	*p* < 0.0001
**Rituximab**			
No Yes Overall	7.9 47.0 33.0	5.000–20.000 25.000–58.000 20.000–58.000	*p* = 0.0023
**DI**			
≥80% <80%	NR 42.0	— 16.000–58.000	**** *p* = 0.1388

**Table 3.** Response rates after first line treatment for 212 elderly DLBCL patients.

Treatment groups	CR #115 (54%)	PR #31 (14.5%)	Stable disease # 8 (4%)	PD # 31 (14.5%)	Non-evaluable # 27 (12%)
**Palliative therapy** **Group 1 # 27** CEPP/PEP-C, # 17 Others # 10 (Steroids SA Rituximab, RT)	**1** 1 0	**1** 1 0	**5** 3 2	**10** 8 2	**10** 4 6
**Non-anthracycline** **Group 2 # 61** Bendamustine-Rituximab, # 8 CEOP+/−R # 39 CVP+/−R, # 9 MVP+/−R # 5	**29** 3**** 21 1 4	**15** 3 9 3 0	**0**	**11** 2 8 0 1	**6** 0 1 5 0
**Anthracycline based** **Group 3 # 124** miniCHOP+/−R, # 36 CHOP+/−R, # 76 da EPOCH-R # 12	**85** 24**** 54 7	**16** 6**** 8 2	**3** 1 2 0	**10** 3 6 1	**10** 2 6 2
